# The circadian clock gates *Drosophila* adult emergence by controlling the timecourse of metamorphosis

**DOI:** 10.1073/pnas.2023249118

**Published:** 2021-06-28

**Authors:** Brandon Mark, Liliana Bustos-González, Guadalupe Cascallares, Felipe Conejera, John Ewer

**Affiliations:** ^a^Centro Interdisciplinario de Neurociencias de Valparaíso, Universidad de Valparaíso, 2360102 Valparaíso, Chile;; ^b^Instituto de Neurociencias de Valparaíso, Universidad de Valparaíso, 2360102 Valparaíso, Chile

**Keywords:** insect emergence, metamorphosis, ecdysone action, eclosion, adult ecdysis

## Abstract

The daily rhythm of adult emergence of holometabolous insects is one of the first circadian rhythms to be studied. In these insects, the circadian clock imposes a daily pattern of emergence by allowing or stimulating eclosion during certain windows of time and inhibiting emergence during others, a process that has been described as “gating.” Although the circadian rhythm of insect emergence provided many of the key concepts of chronobiology, little progress has been made in understanding the bases of the gating process itself, although the term “gating” suggests that it is separate from the developmental process of metamorphosis. Here, we follow the progression through the final stages of *Drosophila* adult development with single-animal resolution and show that the circadian clock imposes a daily rhythmicity to the pattern of emergence by controlling when the insect initiates the final steps of metamorphosis itself. Circadian rhythmicity of emergence depends on the coupling between the central clock located in the brain and a peripheral clock located in the prothoracic gland (PG), an endocrine gland whose only known function is the production of the molting hormone, ecdysone. Here, we show that the clock exerts its action by regulating not the levels of ecdysone but that of its actions mediated by the ecdysone receptor. Our findings may also provide insights for understanding the mechanisms by which the daily rhythms of glucocorticoids are produced in mammals, which result from the coupling between the central clock in the suprachiasmatic nucleus and a peripheral clock located in the suprarenal gland.

## Significance

In holometabolous insects, the circadian clock restricts the time of adult emergence. Although this daily gating of behavior is one of the first circadian rhythms to be studied, little is known about the mechanism underlying the gating process itself. Here, we show that the circadian clock imposes a daily rhythmicity to the pattern of adult emergence by controlling the timing of the completion of metamorphosis. Thus, our findings reveal that the basis of gating is a developmental process and not an acute on/off activational switch and fundamentally changes our understanding of how this circadian control is accomplished. It also provides evidence of a mechanism by which the circadian clock imposes a daily rhythmicity to behavior through the control of the pace of development.

Circadian clocks impose a daily rhythmicity to the behavior and physiology of most multicellular organisms, in which they are believed to provide a mechanism for synchronizing the behavior and physiology of individuals to the daily planetary changes in light and temperature (1). One of the first circadian rhythms to be studied is the rhythm of adult emergence of holometabolous insects (eclosion) (2–4). Eclosion occurs at the end of metamorphosis and is typically restricted to dawn or dusk, depending on the species. Population eclosion profiles show a daily rhythmicity that persists under conditions of constant darkness and temperature and is temperature-compensated, thereby showing the hallmarks of a circadian rhythm (e.g., refs. 4 to 6).

In holometabolous insect species in which this rhythm has been studied, the circadian clock controls the timing of adult emergence by exerting its influence at the very end of adult development. For example, Pittendrigh and Skopik (6) demonstrated in *Drosophila victoria* that overt developmental markers for the progression through metamorphosis, such as the time of eye or bristle pigmentation, occur at times that only depend on the number of hours since the start of metamorphosis and are not affected by changes in the light:dark (LD) cycle. By contrast, eclosion (the final step of metamorphosis) is the only event whose timing is sensitive to the LD regime, with flies always emerging during the morning. Similarly, in *Drosophila melanogaster*, Qiu and Hardin (7) followed the timing of wing pigmentation to show that animals that pigment their wings during the day will primarily emerge during the following day, whereas those that do so at night are delayed an extra ca. 12 h and wait until the light period 2 d later, indicating that the clock exerts its control after the wings have pigmented, during the final day of metamorphosis. Thus, in order to eclose, the insect must have completed metamorphosis and also be within the appropriate time window.

Because the clock intervenes at the end of adult development, the process through which it controls the time of emergence has been described as “gating,” in which emergence is inhibited during certain windows of time and stimulated and/or allowed during others. Although the circadian rhythm of eclosion was one of the first to be studied starting almost 100 y ago (2–4), there is still no clear mechanistic understanding of how this gating process occurs. Nevertheless, so far it has been viewed as a process that is independent of any developmental process. In this scenario, animals that completed metamorphosis prematurely would be prevented from eclosing until the appropriate eclosion gate were opened by the circadian clock (5, 6, 8). However, another possibility is that the clock sets the time of emergence by controlling the timecourse of completion of metamorphosis. Although both scenarios would produce a gated eclosion, they differ mechanistically in fundamental ways. In particular, the first “permissive” gating mechanism predicts that animals could complete metamorphosis at different times prior to emergence and that the clock would then only open or close the window leading to eclosion, depending on the time of the day. By contrast, a “developmental” gating mechanism predicts that animals emerging within a specific gate would speed up or slow down their molt in order to complete metamorphosis in time to emerge during the appropriate gate. In this scenario, gating would be a consequence of the synchronization of metamorphosis. Since in at least some insect species, the titers of the molting hormone, ecdysone (E), express a circadian rhythm (9, 10), a clock control of metamorphosis is a plausible scenario.

Here, we analyzed the timecourse of the final stages of *Drosophila* metamorphosis with single-animal resolution and show that the clock gates eclosion by regulating, primarily, the moment when the final steps of metamorphosis are initiated. We also show that the clock exerts its action by regulating not the levels of the molting hormone itself but that of its actions mediated by the E receptor. Our findings may also provide insights for understanding the mechanisms by which the daily rhythms of glucocorticoid (GC) are produced in mammal, which also depend on the coupling between a central clock, located in the suprachiasmatic nucleus (SCN), and a peripheral clock, located in the suprarenal endocrine gland (11).

## Results

### The Population Assay for Eclosion.

A population assay for eclosion rhythmicity counts the number of flies that emerge over time from a developing population. Under conditions of constant darkness and temperature (DD), wild-type (*per*^*+*^) flies emerge with a periodicity of around 24 h (*SI Appendix*, Fig. S1*A*), whereas flies bearing the *per*^*S*^ allele have a short period clock (12) and produce a strong population rhythm with a periodicity of ca. 20 h (*SI Appendix*, Fig. S1*B*). Finally, flies with a nonfunctional circadian clock (e.g., *per*^*01*^) eclose when they have completed metamorphosis regardless of time of day, producing an arrhythmic composite record (*SI Appendix*, Fig. S1*C*).

The emergence profiles of populations expressing a rhythmic phenotype (e.g., *per*^*+*^ and *per*^*S*^, *SI Appendix*, Fig. S1 *A* and *B*, respectively) imply that each animal contains a functional circadian clock, which ticks with a similar periodicity. This clock then somehow regulates the time of emergence, such that animals that completed metamorphosis at dawn or during the first half of the day (or of their subjective day) will emerge within that circadian gate, whereas animals that did not will then emerge in a subsequent gate. By contrast, the phenotype of populations expressing weaker rhythmicities is much more difficult to interpret. Indeed, a weak population rhythm (e.g., that illustrated by animals of the weakly rhythmic genotype [generic name], *SI Appendix*, Fig. S1*D*) could indicate that every animal has a functional clock but that its period varies significantly within the population; alternatively, it could result from a mixture of a strongly rhythmic subpopulation obscured by a majority arrhythmic one. In addition to not allowing for an interpretation of weak rhythmic phenotypes, this population assay does not allow one to understand how the circadian clock regulates the mechanism that controls emergence so as to cause eclosion to be gated.

### Timecourse of Wing Darkening.

In order to gain insights into the mechanism by which the circadian clock imposes a daily rhythmicity to the pattern of eclosion, we developed a setup (*SI Appendix*, Fig. S2) to follow the timecourse of the progression through metamorphosis of individual flies. For this, we used a custom-built system that produces high-resolution images of flies during the final 2 to 3 d of metamorphosis. The system captures images of up to 100 animals per experiment, taking one image per animal every 12 min (see *Materials and Methods* for details), thereby allowing us to analyze the timing of the process that leads to adult emergence with single-animal resolution.

**Fig. 1.**
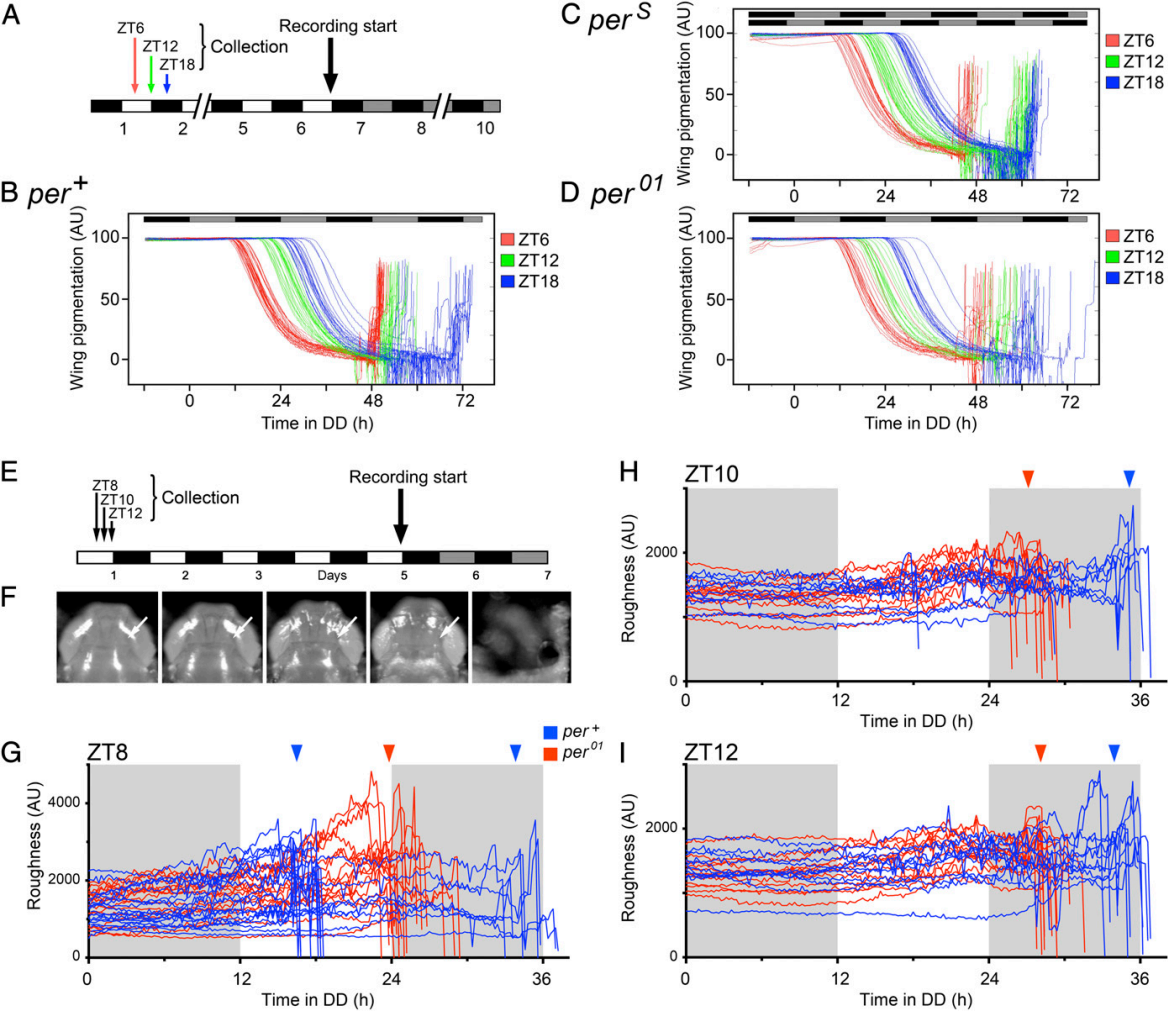
Timecourse of wing darkening (*A*–*D*) and head roughening (*E*–*I*) of individual flies. (*A*) Collection protocol used for results shown in *B*–*D*: white prepupae were collected at ZT6 (red), ZT12 (green), and ZT18 (blue), entrained under 12h:12h L:D for 4 d, then recorded under DD conditions until emergence. (*B*–*D*) Profile of wing darkening for *per*^*+*^ (*B*), *per*^*S*^ (*C*), and *per*^*01*^ (*D*) flies, respectively. Each line shows wing darkening of an individual fly (darker wings produce smaller values) color-coded according to age (collection time, shown in *A*); the sudden increase at the end of each line indicates the moment of emergence. The black/gray boxes indicate the schedule of subjective night and day, respectively; in *C*, the lower schedule is adjusted to this genotype’s ca. 20-h free-running periodicity (*SI Appendix*, Fig. S1*B*, *per*^*S*^). (*E*) Collection protocol used for results shown in *G*–*I*: white prepupae were collected at ZT8, ZT10, and ZT12, entrained under 12h:12h L:D for 4 d, then imaged under DD conditions. (*F*) Changes in the appearance of the head of a single fly during the final 2 d of metamorphosis. Arrows pointing to the area that is initially a single bright area of smooth border (*Left*), which then gradually breaks up into many smaller patches of irregular contour. The final panel shows image of empty puparium, after fly has emerged (*Right*). The time between the leftmost image and emergence (rightmost image) ranged from 24 to 36 h, depending on the genotype and gate within which the fly emerged. (*G*–*I*) Timecourse of head roughening for cohorts of *per*^*+*^ (blue lines) and *per*^*01*^ (red lines) flies of different ages (collection times; shown in *E*); each line represents a different individual fly. The sudden drop at the end of each line indicates the moment of emergence. Gray and white background shading represents subjective night and day, respectively. Blue and red arrowheads indicate the approximate time of emergence of groups of *per*^*+*^ and *per*^*01*^ flies, respectively. Examples of the timecourse of head roughening for individual animals are shown in *SI Appendix*, Fig. S3.

Using this system, we first followed the timecourse of wing darkening (a late marker of development) and the timing of eclosion of groups of flies of different genotypes and ages. For this, we chose three groups of animals that initiated metamorphosis at times separated by 6 h (collected as white prepupae at ZT6, ZT12, and ZT18; Fig. 1*A*; ZT: Zeitgeber time, ZT0: Lights-on; ZT12: lights-off). As shown in Fig. 1 *B*–*D*, it is immediately apparent that wing darkening is a function of developmental time and does not bear a strict relationship with the time of eclosion. Indeed, although the wings of flies with a wild-type clock (Fig. 1*B*) from the three groups pigmented ∼6 h later for each successive group, their emergence (marked by the sudden increase in brightness at the end of every record) was restricted to the subjective day. An especially noteworthy case is that of animals collected at ZT18 (blue traces), which chose to either eclose at the very end of subjective day 3 or at the very beginning of the following subjective day 4. Thus, animals that are of the same chronological age pigment their wings at the same time yet may eclose at times that are as much as ∼12 h apart. A similar situation obtains for *per*^*S*^ animals (Fig. 1*C*), in which adults collected at ZT6 (red traces) eclosed at the end of the gate on subjective day 3 (adjusted for their short 22 h period), whereas those collected at both ZT12 (green traces) and ZT18 (blue traces) eclosed in a staggered manner during the gate on the following circadian day 4. Finally and as expected, the records obtained for arrhythmic *per*^*01*^ flies (Fig. 1*D*) show that flies from the three groups emerged during broad windows separated by ∼6 h. These results are consistent with previous findings (e.g., ref. 7) and show that the process by which the clock gates eclosion is imposed after wing darkening, sometime during the final 12 to 18 h prior to emergence.

### Timecourse of Molting Fluid Resorption: Clock Control of Molting.

In order to investigate how the clock imposes a circadian rhythmicity to the timing of eclosion, we sought markers for the progression through the very final stages of metamorphosis. One such process is the resorption of the molting fluid, which is most apparent in the head, and has previously been used to stage adult animals relative to emergence (13). During metamorphosis, the space between the translucent pupal cuticle and the cuticle of the developing adult fly is filled with molting fluid. Two days before emergence, the surface of the pupal cuticle surrounding the head presents a smooth surface due to the amount of molting fluid contained between the two cuticles and produces a single bright patch of reflection when illuminated with oblique illumination (Fig. 1*F*, arrow leftmost panel; far red illumination was used for these experiments, to which the circadian clock is blind) (14). As the end of metamorphosis approaches, the molting fluid is gradually resorbed, causing the pupal cuticle to become progressively rougher as it now sticks to the irregular surface of the head and the eyes. This change is evident as a gradual breaking up of the initial patch of reflection, which initially has a smooth outline, into an increasing number of smaller patches of irregular border (Fig. 1*F*, arrow, *Left* to *Right*; Movies S1 and S2) and culminating with the emergence of the fly (Fig. 1 *F*, *Right*). We produced a “roughness index” by computing either the sum of the perimeter of these patches or the sum of the SD between the intensity at each point of the image and that of the pixels immediately surrounding it. Results using either measure were similar and generally showed (Fig. 1 *G*–*I*) that roughness increased during the final few hours prior to emergence, which is marked by the sudden drop in roughness at the end of each record and which shows the appropriate gating (refer to *SI Appendix*, Fig. S3 for traces for a few individual animals). As expected, the records for wild-type flies showed the expected gating of eclosion (marked by blue arrowheads in Fig. 1 *G*–*I*), with some flies (e.g., those collected at ZT8, Fig. 1*G*) even splitting such that some emerged around noon of the first subjective day whereas the remainder emerged during the second gate, which opened at (subjective) dawn. By contrast, arrhythmic animals emerged at times separated by ca. 2 h (time between collection times; red arrowheads in Fig. 1 *G*–*I*). The influence of the clock is also apparent in the fact that wild-type flies completed metamorphosis either earlier than did arrhythmic ones or did so later so as to emerge during permitted windows of time.

**Fig. 2.**
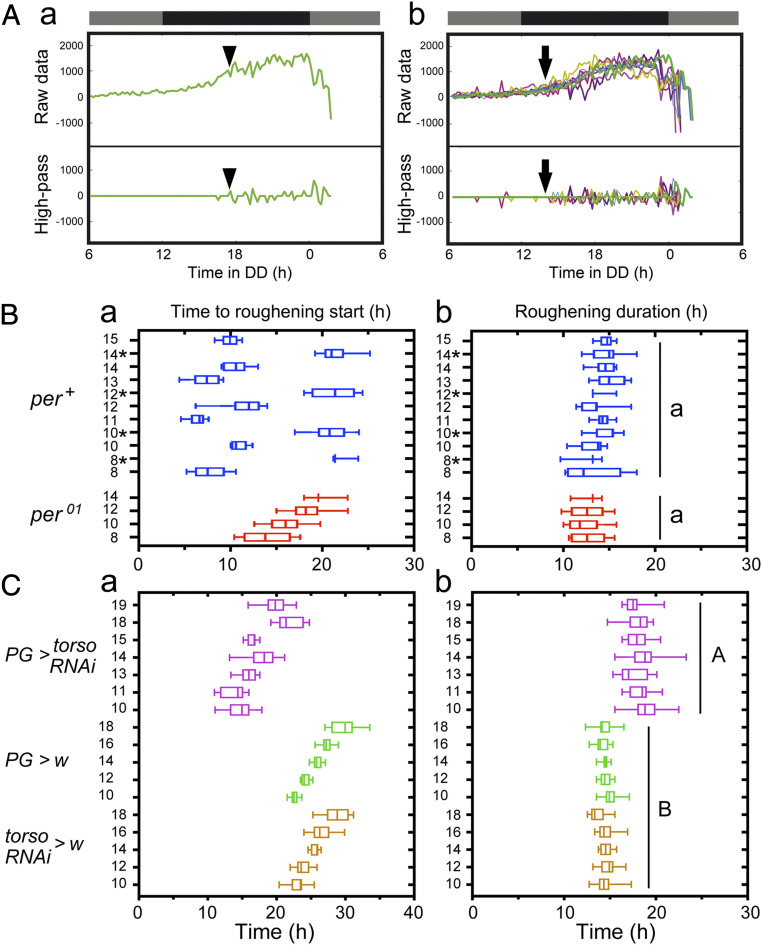
Timecourse of head roughening. (*A*) Raw (*Top*) and high-pass filtered (*Bottom*) records of the timecourse of head roughening of an individual fly (*a*) and of a group of flies (*b*) of the same age and genotype. Arrowheads (in *a*) and arrows (in *b*) indicate the moment when the process of roughening started. (*B*) Average time (indicated as box plots) for the start of head roughening (*a*) and the duration of head roughening (*b*) for groups of *per*^*+*^ (blue symbols) and *per*^*01*^ (red symbols) flies of different ages (i.e., collected at different times, indicated along *y*-axis). Groups of *per*^*+*^ flies of the same age which chose a later emergence gate are indicated with “*”. (*C*) Average time (indicated as box plots) for the start of head roughening (*a*) and the duration of head roughening (*b*) for flies bearing knockdown of *torso* in the PG (magenta symbols) and for relevant controls (green and yellow symbols). In *B*, *a* and *C*, *a*, “Time to roughening start” corresponds to the number of hours between the start of DD and the start of head roughening. In *B*, *b* and *C*, *b*, “Roughening duration” is the number of hours between the start of the head roughening and the time of emergence; different letters indicate statistically significant differences (*P* < 0.05; one-way ANOVA, Tukey post hoc analysis). For *B*, *n* = 7 to 10 per genotype per time and group; for *C*, *n* = 10 to 30 per genotype and time.

Although the amplitude and timecourse of the changes in roughness were quite variable between individuals, we noticed that there was a specific moment when roughening started, which could be detected by applying a high-pass filter to the timecourse of roughening (Fig. 2 *A*, *a*, *Lower*; *SI Appendix*, Fig. S3). Furthermore, the moment when this occurred was relatively consistent across animals of the same genotype and developmental age (Fig. 2 *A*, *b*, *Lower*). We then investigated the relationship between the time of start of roughening and emergence. We were especially interested in those cohorts of animals that were of the same chronological age yet emerged over two separate gates (*cf* Fig. 1*G*). As shown in Fig. 2 *B*, *a*, groups of wild-type (*per*^*+*^) flies (blue symbols) of the same age that emerged in the later gate (indicated by “*” in Fig. 2 *B*, *a*) started roughening ∼14 h later than did those that chose the earlier gate. Since the process that is being followed is a readout of the progress through metamorphosis, these results show that in contrast to what obtains for wing darkening (e.g., Fig. 1*B*), animals of the same age that emerged in different gates started the final steps of the molt at different times. As expected, *per*^*01*^ (arrhythmic) flies of different ages started roughening at progressively later times (Fig. 2 *B*, *a*, red symbols). Of note, wild-type animals of different ages that chose to eclose late within the first gate started roughening at around the same time, whereas arrhythmic animals of the same age did so at progressively later times. Interestingly, we found no differences in the duration of the roughening process itself, regardless of the fly’s genotype (Fig. 2 *B*, *b*). Thus, the circadian clock appears to regulate the time when the animal commits to complete metamorphosis. By contrast, the time required to do so is independent of the gate and time of emergence.

In order to explore the utility of this assay, we examined the timecourse of roughening in animals of a different genotype that produces an arrhythmic population emergence record. In *Drosophila*, the receptor to the PTTH neuropeptide, which regulates steroidogenesis by the prothoracic gland (PG), is encoded by the *torso* gene (15), and we have previously shown that knockdown of *torso* in the PG renders arrhythmic the pattern of adult emergence (16). Fig. 2 *C*, *a* (magenta symbols) shows that the timecourse of roughening onset in these animals is similar to that of *per*^*01*^ animals (Fig. 2 *B*, *a*, red symbols) (note the difference in scale of the *x*-axis), becoming progressively later in animals of older chronological age (the relevant controls, Fig. 2 *C*, *a*, green and yellow symbols, all emerged within the second gate due to the collection times selected and could thus emerge progressively later yet remain within the same gate). Strikingly, however, animals bearing *torso* knockdown in the PG roughened significantly more slowly that did their corresponding controls (compare Fig. 2 *C*, *b*, magenta versus green and yellow symbols). This is consistent with the fact that *torso* is involved in regulating the molting process itself and that knockdown of *torso* is known to significantly lengthen the third and last larval instar (15). This example reveals that this assay can be used to investigate the mechanism that controls the timing of emergence.

### Role of E Signaling in Regulating the Timing of Emergence.

Our results indicate that the circadian clock gates emergence by committing the animal to complete metamorphosis. Since emergence requires the titers of the molting hormone, E, to drop below a threshold level (e.g., refs. 17 to 19), one possible mechanism mediating this control would be for the clock to control the titers of E, a mechanism that may apply for the hemimetabolous insect, *Rhodnius prolixus* (20). Nevertheless, such a mechanism seems unlikely in the case of *Drosophila*, because ecdysteroid levels are extremely low during the 20 h prior to emergence (ca. 10 pg/animal), and there is no evidence that the titers show any circadian changes during the period starting 24 to 12 h before emergence (21, 22). Notwithstanding this evidence, we explored the relationship between the titers of 20E (the active form of E) and the time of emergence by determining the effects of injecting 20E on the time of emergence. If 20E titers were regulated by the clock during the final stretch of metamorphosis, then injecting increasing 20E prior to emergence should disrupt the gating and cause a gradual delay in the time of emergence. Such a result was obtained in *Rhodnius*, supporting the hypothesis that in this insect, the clock regulates gated molts by controlling 20E titers (20).

**Fig. 3.**
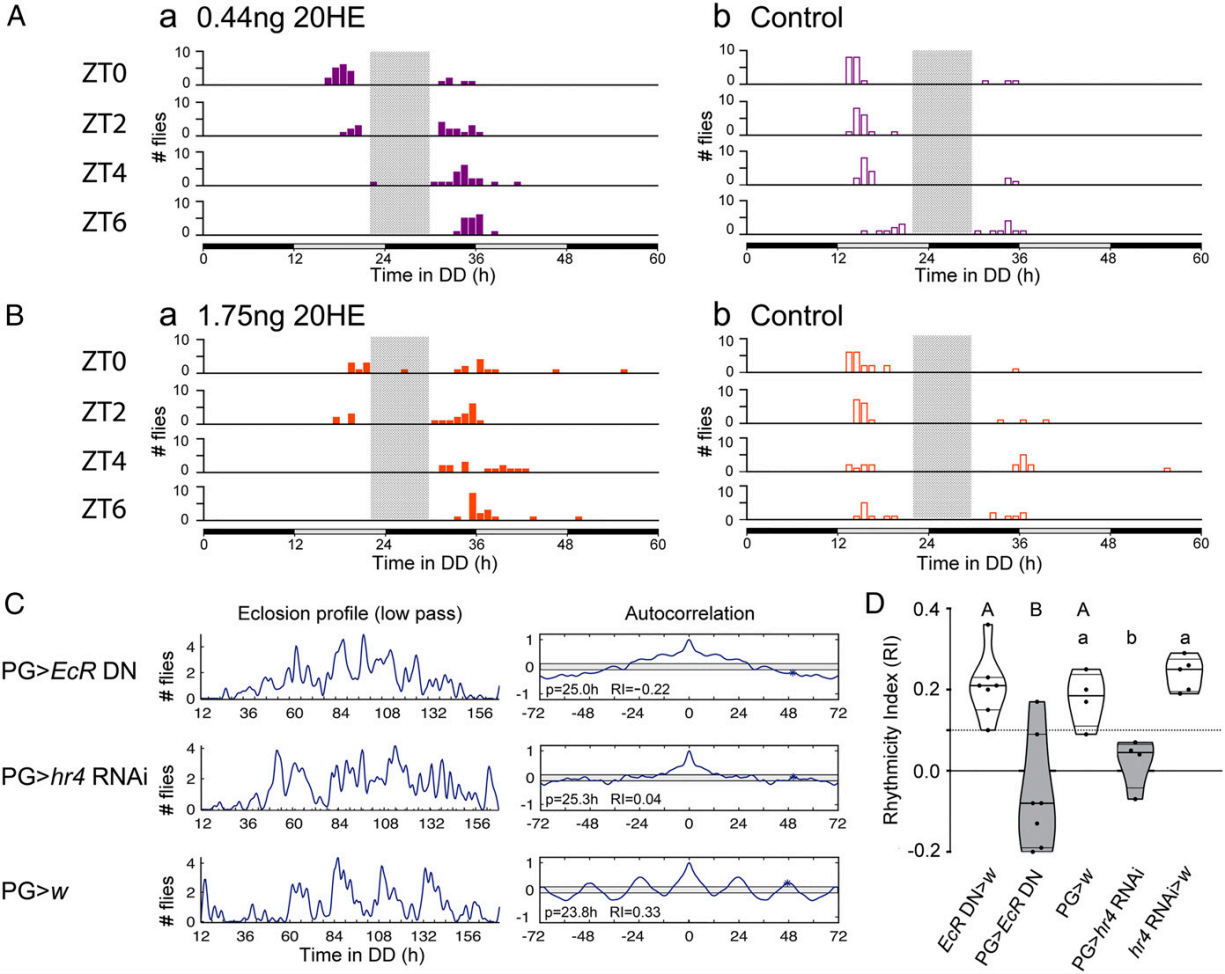
Role of 20E and EcR in the gating of emergence. (*A* and *B*) Effect of injections of 0.44 ng (*A*) and 1.75 ng (*B*) of 20E (*Left*) and corresponding vehicle control (*Right*) on the time of emergence. Gray bar indicates period of time during which emergence is prohibited. Black/gray boxes along the *x*-axis indicate schedule of subjective night and day, respectively. Injections were done around 2 h before lights-off on the day prior to the start of recording (∼16 h before predicted eclosion). (*C*) Profile of emergence (*Left*) and corresponding autocorrelogram (*Right*) of population of animals expressing a dominant form of EcR (EcR DN) or bearing a knockdown of *hr4* in the PG and that of relevant controls; periodicity (p, in hours) and RI are indicated within frame. (*D*) Corresponding summary of RI represented as violin plots. Each dot represents a different experiment; the dashed line at RI = 0.1 indicates cutoff below which records are considered arrhythmic. Different letters indicate statistically significant differences (*P* < 0.05; one-way ANOVA, Tukey post hoc analysis).

Fig. 3 *A*, *a* and *B*, *a* show the timing of emergence of staged animals injected 16 to 22 h before their expected time of emergence with 0.44 ng (Fig. 3 *A*, *a*) or 1.75 ng (Fig. 3 *B*, *a*) of 20E and that of their respective controls (injection of corresponding volume of solvent; Fig. 3 *A*, *b* and *B*, *b*). As expected, injections of 20E delayed the time of emergence. Thus, for example, as shown in Fig. 3*A*, most animals that started metamorphosis at ZT2 and ZT4 and were injected with 0.44 ng 20E emerged at the start of the second day, whereas controls (injection of solvent only) emerged mostly during the preceding day. In addition, animals injected with the higher 1.75 ng dose of 20E (Fig. 3 *B*, *a*) showed a greater delay than did those injected with 0.44 ng (Fig. 3 *A*, *a*). Most importantly, however, in all cases a gating was still observed (indicated by gray bar in Fig. 3 *A* and *B*). For example, animals injected with 0.44 ng 20E (Fig. 3 *A*, *a*) avoided emerging during the late afternoon of Day 1 and the early night of Day 2, emerging either during the afternoon of Day 1 or the early morning of Day 2. A similar result was observed with the higher dose of 1.75 ng (Fig. 3 *B*, *a*). Injections with the highest doses tested, 7 ng, were inconclusive because they caused high levels of mortality, likely because they disrupted metamorphosis itself. Thus, whereas injections of 0.44 and 1.75 ng caused around 10% lethality (*N* = 77 and 85 animals injected, respectively), injections of 7 ng caused 76% lethality (*N* = 89 animals injected); lethality for controls was 5 to 10% for all doses, with 60 to 70 animals injected per dose.

Consistent with findings reported for the flesh fly, *Sarcophaga crassipalpis* (23), and the moth, *Manduca sexta* (24), our results show that 20E injections delay the timing of emergence without disrupting its gating. It is nonetheless surprising that injecting 20E on the day before emergence can exert measurable delays on the timing of eclosion. Indeed, 20E levels are extremely low during the 20 h prior to emergence [ca. 10 pg/animal (21, 22)], and most cells of the PG have undergone cell death starting 30 to 40 h after puparium formation (25), which might have implied that 20E is no longer a biologically significant signaling system at that time.

Thus, our results suggest that the mechanism by which the circadian clock gates the timing of emergence intervenes downstream of 20E, in the transduction pathway of 20E action. In order to explore this possibility, we determined the consequences on the timing of emergence of expressing in the PG the dominant form of the 20E receptor, EcR (EcR DN). We also down-regulated *DHR4*, an E-induced nuclear receptor that is a component of the E transduction pathway and has been suggested to act as a mediator of EcR actions during metamorphosis (26, 27). As shown in Fig. 3 *C* and *D*, disrupting 20E signaling in the PG rendered arrhythmic the pattern of emergence.

The arrhythmicity caused by interfering with *EcR* and *dhr4* function suggests that these genes play a key role in the mechanism by which the circadian clock imposes a daily rhythm to the pattern of adult emergence. However, 20E and EcR play a vital role during *Drosophila* development and molting. Thus, the arrhythmicity observed following knockdown of EcR during the entire life of the fly might be an artifact due to developmental defects caused by the sustained reduction in 20E signaling. In order to determine whether rhythmic emergence required EcR function at the end of metamorphosis, we used the temporal and regional gene expression targeting (TARGET) system (28) to restrict EcR knockdown to the final stages of adult development. This system relies on the ubiquitous expression of a temperature sensitive version of the GAL4 inhibitor, GAL80 (*tubulin*-GAL80[ts]). Thus, we used this system to restrict expression of EcR DN in the PG to the second half of metamorphosis by raising the animals at low temperature (20 °C), then increasing it to 30 °C starting shortly before adult emergence. This temperature change would relieve the inhibition caused by GAL80, thereby allowing expression of EcR DN in the PG. Although this is currently the best method available to temporally restrict gene expression in *Drosophila* pupae, it has, in the context of these experiments, two inherent drawbacks. The first is that it takes several hours for de-repression to result in normal levels of gene expression. Although we do not know the timecourse for EcR DN de-repression, preliminary experiments using a green fluorescent protein (GFP) reporter revealed that readily visible levels of fluorescence were not apparent in the PG of developing adults for ∼24 h after transferring the animals to high (permissive) temperatures. Interestingly, in developing adults, fluorescence also took longer to increase and reached lower levels than what was observed in third instar larvae. The second drawback is that 30 °C greatly accelerates *Drosophila* development, causing eclosion records (obtained with the population assay used here) to never be spread out over sufficient days to allow for the quantitative analysis of periodicity. Nevertheless, a key feature that distinguishes rhythmic from arrhythmic records is that the former invariably includes valleys spaced by ∼24 h, during which few flies emerge and which reflect the presence of times when emergence is not permitted (e.g., Fig. 3*C*, PG > w control; and *SI Appendix*, Fig. S1, A- *per*^*+*^ versus C-*per*^*01*^). Using this criterion for rhythmicity, our results (*SI Appendix*, Fig. S4) show that knockdown of EcR during the second half of metamorphosis is sufficient to cause arrhythmicity, suggesting that 20E signaling is required at the end of metamorphosis in order for emergence to express circadian rhythmicity.

**Fig. 4.**
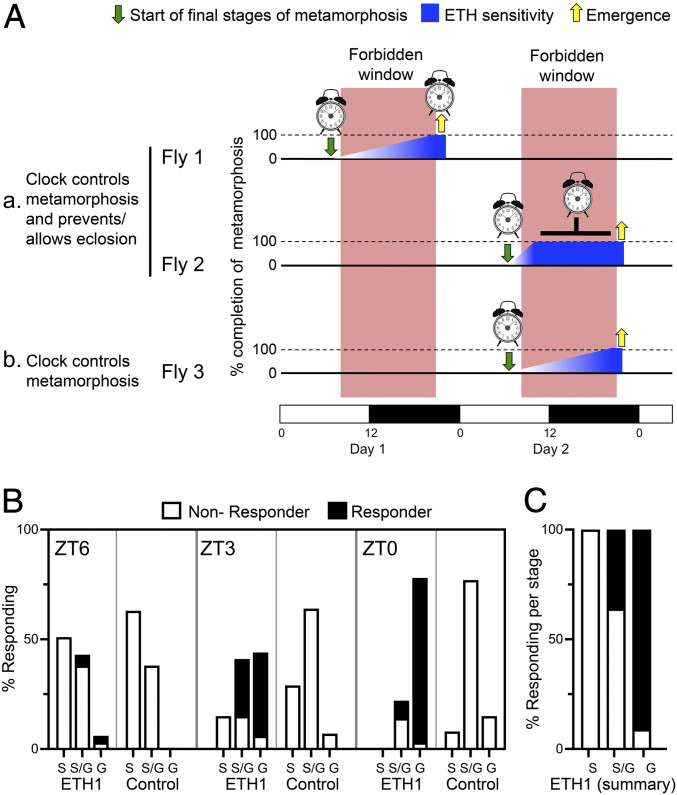
Role of the circadian clock in the control of the timing of eclosion. (*A*, *a*) The clock controls the time at which the fly initiates the final steps of metamorphosis and either allows emergence to occur when metamorphosis is completed within a gate (Fly 1) or prevents the emergence of animals that completed metamorphosis within a forbidden window (Fly 2). (*A*, *b*) The clock controls the time at which the fly initiates the final steps of metamorphosis (Fly 3). Blue wedge symbolizes the onset of ETH sensitivity. (*B* and *C*) ETH injections cause premature emergence only in animals that have reached the end of metamorphosis. Histograms in *B* indicate the proportion of animals that were at the “S,” “S/G,” or “G” stage within each group and either eclosed (responders, filled bars) or did not eclose (nonresponder, open bars) following ETH injections. The number of animals in each group was the following: ZT6: ETH: 39; control 16; ZT3: ETH: 34; control 14; ZT0: ETH: 36; and control 13. (*C*) The results obtained in *B* following ETH injection, tabulated as a function of developmental stage.

**Fig. 5.**
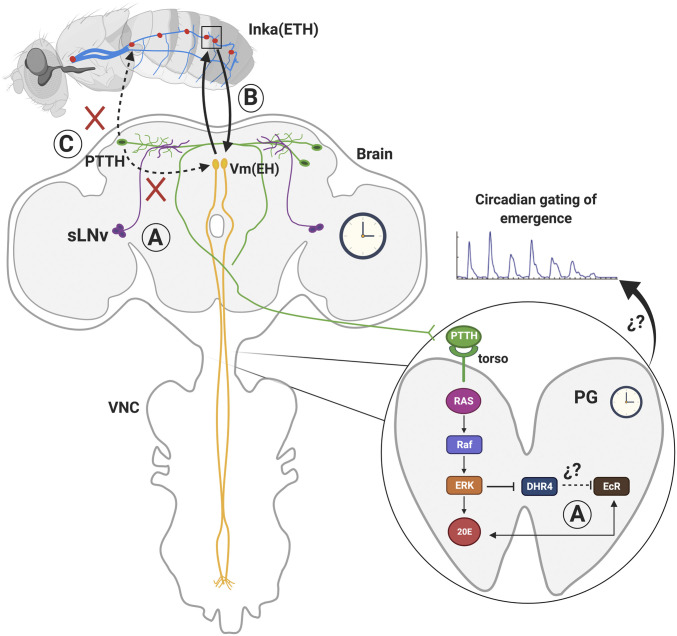
Proposed model for clock control of the timing of emergence. Schematic depicting the neuronal and cellular circuits and molecular components of the brain (*Left*) and PG clock (*Right*) that could be involved in the circadian regulation of the emergence behavior is shown. (*A*) sLNv clock neurons (purple) project dorsally and are known to transmit time information to PTTH neurons (green). PTTH acts on the PG clock via its receptor, TORSO. The intracellular transduction pathway activated by PTTH regulates DHR4 signaling, which in turn can act as an E-induced repressor of EcR, influencing 20E action. (*B*) Endocrine-positive feedback between ETH-containing epitracheal cells (Inka cells, red) and EH-containing neurons (Vm neurons, yellow) that turns on emergence. Yet, Vm neurons are not part of the mechanism by which the circadian clock regulates the timing of eclosion (red crosses) because targeted ablation of Vm neurons does not abolish the circadian rhythmicity of emergence. (*C*) PTTH itself could not transmit the time signal directly from the brain to nonclock cells that turn on the ecdysis behavior (Inka cells and Vm neurons) because *torso* expression is required only in the PG for a circadian rhythmicity of emergence.

So far, our results show that the circadian clock regulates the time when the final stages of metamorphosis are initiated (green arrows in Fig. 4*A*) and that the duration of this final segment of adult development is constant. Yet, this scenario does not preclude the possibility that the clock also prevents the eclosion of animals that have completed metamorphosis prematurely, during the “forbidden period” (“Fly 2” in Fig. 4 *A*, *a*). In order to explore this possibility, we used Ecdysis Triggering Hormone (ETH) injections to determine whether there were animals that had completed metamorphosis while the eclosion gate was closed and were prevented from eclosing due to the inhibition by the clock. ETH together with Eclosion Hormone (EH) are the neuropeptides that turn on the ecdysial motor program, including that of adult emergence (29–31) (*cf* Fig. 5*B*). Thus, ETH injections would bypass clock control and cause the emergence of animals that were developmentally able to eclose but prevented by the clock from emerging during a forbidden window of time.

For these experiments, we collected white prepupae at three different times separated by 3 h and entrained them using the LD and temperature regime used in previous experiments (see *Materials and Methods*). Five d later and ∼9, 6, and 3 h before emergence, we scored the pharate adults of all groups for their developmental proximity to emergence using the developmental markers used by Kimura and Truman (13) and evaluated their response to injections of ca. 1 pmol synthetic ETH versus solvent alone. In such experiments, animals that are sensitive to ETH eclose within 2 h of injection [called “Responders” (31)], whereas those that are not will eclose later as a result of the release of their endogenous stores of ETH (called “Non-responders”).

The results of these experiments are summarized in Fig. 4*B* and show that the fraction of animals that responded to ETH increased in chronologically older cohorts of animals. Indeed, a greater fraction of animals responded in the group collected at ZT0 versus ZT3 (ZT0: 30 out of 36 versus ZT3: 22 out of 34), which in turn contained a greater fraction of animals responding than did the cohort of animals collected at ZT6 (ZT3: 22 out of 34 versus ZT6: 3 out of 39). Importantly, however, animals from older groups were, naturally, also more developmentally advanced. Indeed, in later cohorts, the percentage of animals scored as “Grainy” [G; around 3 h before eclosion, (13)] increased, whereas that of animals classified as “Smooth” (S; around 9 h before eclosion) decreased. Thus, the increase in responsiveness to ETH in later cohorts simply reflected the fact that they contained animals that were further along in their adult development. This is quantitated in Fig. 4*C*, which shows that, consistent with Park et al. (31), most animals at the “G” stage responded to ETH, whereas none of those at the “S” stage did so, with those at the intermediate “S/G” stage showing an intermediate responsiveness. Thus, we found no evidence for the existence of animals that had completed metamorphosis prematurely and were prevented from emerging by inhibition from the circadian clock. Indeed, the only animals that responded to ETH injections were those that were within 2 to 3 h of emergence and had become responsive to this hormone. These results suggest that the clock gates eclosion primarily by controlling the time when the final stages of metamorphosis are initiated (“Fly 3” in Fig. 4 *A*, *b*).

## Discussion

The daily rhythm of adult insect emergence was one of the first circadian rhythms to be studied and contributed significantly to our understanding of aspects of chronobiology, including temperature compensation, phase response curves, and responses to skeleton photoperiods among others (4). It also provided the assay that led to the isolation of the first mutations affecting animal circadian rhythmicity and defined the *period* (12) and, later, the *timeless* (32) genes, which were the key entry points that eventually led to our understanding of how circadian rhythms are produced in animals (33). Yet, for all its importance, our understanding of how the circadian rhythm of emergence is produced remains descriptive and is framed in terms of a gating process. This apt description defines periods of the day (and subjective day) within which the circadian clock allows animals to emerge and others within which emergence is prohibited. Framing the role of the clock as “gating” suggests that the clock imposes a daily rhythm to adult emergence by turning on the emergence process during the gate and preventing it from turning on once the gate is closed; importantly, it assumes that the clock plays only a permissive role, either stimulating or inhibiting emergence, which would be separate from the process of metamorphosis itself. Such a mechanism could be mediated by stimulating the neurons that produce the neuropeptides that control emergence behavior, thereby opening the gate, or inhibiting them to keep it closed (Fig. 5*A*). Although this scenario is anatomically plausible (e.g., refs. 34 and 35), targeted ablation of the principal EH- (36) and CCAP-containing neurons (34), which play key roles in the control of ecdysis (19, 37) does not eliminate the circadian rhythmicity of eclosion, although it does eliminate the “lights-on” response (34, 38), a burst of emergence triggered by the lights-on transition that occurs under a LD regime.

The assumption that the circadian clock gates emergence by activating an on/off switch at the appropriate times implies that animals fated to eclose within a given gate could complete metamorphosis several hours before the gate opened. However, the population assay that is used to monitor the rhythmicity of emergence does not allow this key prediction to be tested. Here, we developed an assay that allows the progression through the final stages of adult development to be monitored with single-animal resolution. Our findings suggest that the circadian clock gates emergence by controlling the time when the animal commits to complete metamorphosis. In addition, using ETH injections, we found no evidence for the existence of animals that had completed metamorphosis prematurely and were prevented from emerging by inhibition from the circadian clock. Thus, these results show that the clock controls when the final steps of metamorphosis are initiated and appears to not intervene in subsequent events.

A rhythmic pattern of emergence requires functional clocks in the brain and in the PG (16, 39–41), an endocrine gland whose only known function is the production of E, the precursor of the bioactive molting hormone, 20E (42) (Fig. 5*B*). However, and consistent with findings from other holometabolous insects (23, 24), we found that the clock does not gate eclosion by controlling the levels of 20E. Instead, we found that this control is affected downstream of 20E, at the level of the 20E receptor, EcR. An analogous situation has been reported in mammals, in which the daily GC rhythm results from the coupling between the central clock in the SCN and the peripheral clock housed in the suprarenal gland (11, 43). In this case, the SCN regulates the GC rhythm via the hypothalamic–pituitary–adrenal axis as well as the autonomic nervous system. For its part, the suprarenal gland contributes to the GC rhythm by expressing circadian changes in responsiveness to ACTH. Yet, the bases for these changes of sensitivity are not fully understood, and it is here that our findings on the role of the brain and the PG clock in regulating emergence may provide useful hypotheses for understanding how the circadian rhythmicity in GC titers is produced.

How might the clock control 20E action? The transcriptional responses triggered by the steroid, 20E, are mediated by its nuclear hormone receptor, a heterodimer comprised of EcR and ULTRASPIRACLE, that acts by coordinating the expression of downstream genes, many of which are themselves hormone and/or nuclear receptors, including EcR itself (44). Although *E75*, a 20E-induced gene member of the nuclear receptor superfamily, is known to interact with core elements of the circadian clock (45, 46), there are no reports documenting how the clock might, in turn, directly influence 20E action. One possible route could be via the nuclear hormone receptor, *DHR4*. *DHR4* can act as an E-induced repressor of EcR (26), and its cytoplasmic-versus-nuclear localization is regulated by the neuropeptide, PTTH, and its receptor, *torso* (27). Thus, although PTTH plays a key role in the control of steroidogenesis by the PG (47), its regulation of *DHR4* signaling could provide a route by which the brain clock could influence 20E action (Fig. 5, within PG). Consistent with this hypothesis, knockdown of *dhr4* in the PG eliminated the rhythmic pattern of emergence (Fig. 3 *C* and *D*), although the requirement for *DHR4* function specifically during the second half of metamorphosis remains to be investigated because experiments carried out with available genetic tools used to restrict *dhr4* knockdown starting at the end of metamorphosis were inconclusive. Another possible route for the clock to influence 20E action could be through the regulation of EcR intracellular localization. In vertebrates, the core clock gene, *clock*, regulates the transcriptional activity of the GC receptor by acetylating lysine residues located in its hinge region (48), which include a nuclear localization signal (NLS). Interestingly, similar NLSs are present in the hinge region of several members of the nuclear receptor superfamily including EcR (49). Regardless of how the clock may control EcR action, simply maintaining EcR signaling at the levels reached at the time of wing pigmentation might be sufficient to stop the progress of metamorphosis. Indeed, it is well known that 20E infusions will keep in “suspended animation” animals undergoing metamorphosis (e.g., ref. 50), and a similar outcome would, presumably, be obtained by controlling the levels of 20E signaling at that time. Although we do not yet understand how the clock might control the levels of 20E signaling, our findings do indeed suggest that gating depends on 20E signaling via its receptor, EcR.

Downstream of the PG, we do not currently understand how the circadian signal would be transmitted from the PG to the relevant neurons and cells that control the execution of emergence behavior. Since the clock does not transmit this information via 20E, a different signal must be involved. One possible candidate is again, PTTH, which could, via *DHR4*, modulate the response of target cells to 20E in a time dependent fashion (Fig. 5*C*). However, we recently showed that, of the cells that express the clock gene, *timeless*, the PTTH receptor, *torso*, is only required in the PG for a circadian rhythm of emergence (16). Thus, if PTTH were mediating this function, it would need to transmit the time signal from the brain to nonclock cells. In addition, proposing that PTTH would transmit the time signal directly from the brain clock would bypass the known requirement for a functional clock in the PG for circadian rhythmicity of emergence. A final complication to this hypothesis is that PTTH expression appears to not be circadian but show a ca. 8 h periodicity (51).

We also do not understand how the clock would regulate the moment when emergence behavior is turned on. The signal that turns on each ecdysis, including adult emergence, depends on an endocrine-positive feedback between the neuropeptides, ETH and EH (29, 30) (Fig. 5*B*). Supplies of these neuropeptides are fully replenished by the end of the molt; thus, activating the ecdysis motor program depends on the presence of responsive ETH and EH receptors (ETHR and EHR, respectively) in target cells and an initial release of either ETH or EH. In moths, sensitivity to ETH depends on the rise in 20E titers that occurs at the start of the molt, whereas sensitivity to EH depends on the fall of 20E to very low levels (37), which, presumably, could also be accomplished by reducing EcR action. Yet, preliminary analyses of the upstream region of the putative EHR gene [encoded by the CG10738 gene (52)] did not identify perfect matches to the known canonical EcR binding motifs (53, 54), making the simplest version of this hypothesis unlikely.

In summary, we have shown here that the circadian clock imposes a daily rhythmicity to the pattern of adult emergence by controlling when the final steps of metamorphosis are initiated. Our findings reveal that the basis of gating is a developmental process and not an acute on/off activational switch and fundamentally changes our understanding of how this circadian control is accomplished. Future work will be tasked with identifying the mechanism by which the clock controls the time when the final steps of metamorphosis are initiated and determine how this signal is then transmitted to the mechanism that controls emergence behavior.

## Materials and Methods

### Fly Stocks.

Flies were raised on standard cornmeal/yeast media and maintained at room temperature (20 to 22 °C) under a 12h:12h LD schedule. Stocks of *period* (*per*) alleles (*per*^*+*^, *per*^*01*^, and *per*^*S*^) were obtained from Jeff Hall. The *phm*-GAL4 driver was obtained from Michael O’Connor and drives expression in the PG. As shown in *SI Appendix*, Fig. S5, we were able to detect reporter expression in some additional tissues of pharate adults. Yet, these sites are likely not relevant for circadian rhythmicity (*cf* *SI Appendix*, Fig. S6). The following Upstream Activation Sequence-RNA interference (UAS-RNAi) stocks were obtained from the Vienna *Drosophila* Resource Center (VDRC): UAS-*torso* RNAi (VDRC109108) and UAS-*dhr4* RNAi (VDRC #37066). They were always used in combination with UAS-*dcr2* (obtained from the Bloomington *Drosophila* Stock Center) to potentiate RNAi-mediated knockdown. The UAS-EcR dominant negative transgene was UAS-EcR.B2.F645A. Stocks bearing this insert as well as all other stocks were obtained from the Bloomington *Drosophila* Stock Center.

### Production of Isogenic Period Stocks.

The genetic background of stocks bearing different *per* alleles was isogenized through repeated crosses to the same *yellow*, *white* (*y*,*w*) stock (the *white* and *period* genes are adjacent on the X chromosome; thus, these two genes almost invariably cosegregate). For this, three to five males for each *per* genotype and also from *FM7a* balancer stock were crossed separately to five to eight *y*,*w* virgin females. Heterozygous virgin female progeny were then crossed to *y*,*w* males, and the process repeated for 10 generations. Resulting heterozygous virgin females were then crossed to *FM7a* males; progeny flies were used to then produce a homozygous stock for each of the three *per* genotypes. Two lines were isogenized in parallel for each *per* allele to control for effects that did not map to *per*; their genotype was confirmed using locomotor activity testing at the end of the isogenization process. Flies from both lines were used for the experiments described here.

### Locomotor Activity Assay.

For each genotype, eight to 10 females plus four to five males were reared at 20 °C under LD 12:12 cycles (lights-on at noon). Male progeny were collected under CO_2_ anesthesia 1 to 2 d after eclosion, aged and entrained 5 d, then placed in TriKinetics activity monitor at 20 °C. Their activity was then monitored under DD conditions for 10 d.

### Population Assay for Eclosion.

For each culture, 50 females plus 10 males were raised at 20 °C under LD 12:12. Resulting pupae were collected (typically from four to eight cultures), fixed onto eclosion plates with Elmer’s glue, mounted on TriKinetics eclosion monitors (TriKinetics), and entrained for 3 to 4 d. Emergence was then monitored under DD for 7 to 10 d at 20 °C in climate-controlled chambers with ca. 65% relative humidity (Bioref 19L incubator, PiTec). For experiments using *tub*-GAL80[ts], flies were raised at 20 °C and entrained under LD as describe above. The resulting pupae were placed in eclosion monitor as previously described and the temperature raised to 30 °C on the day before the oldest pupae would start emerging and kept at this temperature until the end of the experiment. Rhythmicity was evaluated using a MATLAB-based analysis software package (55). The strength of rhythmicity was quantified using the rhythmicity index (RI) derived from autocorrelation analysis. By this measure, records are considered rhythmic when RI is greater than 0.3, weakly rhythmic for RI values in the range of 0.1 to 0.3, and arrhythmic when RI is less than 0.1 (or when record is obviously aperiodic) (56).

### White Pupa Collection.

In total, 50 to 100 females and males were placed on fly media in bottles and discarded after 24 h. Resulting white prepupae were collected at the desired times (for consistency and slightly larger size only female animals were used) and placed in Petri dishes on humidified paper and maintained in a Percival incubator with 12:12 LD schedule and a ca. 22 °C(L):20 °C(D) temperature cycle. This small daily temperature cycle was used for entrainment in addition to the LD cycle in the hope that it would improve the synchronization among animals, as has been observed for other insect species (57). The exact temperature was monitored and recorded using iButtons (Maxim Integrated). The white prepupal period lasts only ca. 15 min; thus, animals collected at this stage represent a tightly synchronized cohort. For each experiment, a complete set of genotypes was collected (genotypes to be tested and relevant controls) and tested in parallel. The only exception was for experiments in which *torso* was knocked down in the PG, for which experimental animals were significantly larger than were controls. In this case, the two groups were imaged in successive experiments.

### Filming Setup.

On day 4, up to 100 previously collected pupae were transferred from Petri dishes to a 12-cm diameter Plexiglas filming disk and placed on double-stick tape along a line etched on the disk; groups were separated with small strips of red tape. Humidity was maintained with humidified paper towels, and a large Petri dish was used as cover. On day 5, the operculum of all the animals was removed, close to the time of lights-off. A piece of humidified paper towel was placed in the center of the disk as well as an iButton (Maxim Integrated), used to monitor temperature. The disk was then covered with an inverted large Petri dish whose base had been replaced by glass to increase transparency. The disk, which was mounted on an alternating current (AC) timing motor (Hansen Motor Corporation) that turned 5 times per h, was then placed under a Leica MZ95 dissecting microscope in such a way that the animals passed under the field of view of the microscope as it turned. After lights-off, the temperature was maintained at the night temperature, and white lights were kept off for the duration of the experiment. During the experiment, animals were illuminated using (2) 840-nm light-emitting diodes (LEDs) (LED840-04AU; Roithner Lasertechnik) and imaged through the dissection microscope using a Watec infrared camera (WAT-902H ultimate; Watec).

### Image Capture.

Images were captured using a PCI-1410 National Instruments Analog/Digital frame grabber (National Instruments Corporation). The capture of 10 images was triggered as soon as the average light intensity of two central regions of the image exceeded threshold values, resulting in 10 successive images being captured per animal per timepoint. Images were captured until all animals had emerged (typically 24 to 36 h) and stored on an external hard drive. Because the disk on which the flies were mounted turned 5 times per h, a burst of 10 images was captured for each animal every 12 min. An in-house–made script was then used to reconstruct the time series for each animal by selecting the most centered image of each fly from the set of images captured every 12 min. Script data have been deposited in GitHub (https://github.com/johnewer/DMM_Analisis).

### Image Analyses: Wing Darkening.

The timecourse of wing darkening was measured using an in-house–made script. For this, the time series for each animal was first stabilized and aligned to eliminate any small jitter that might have occurred between successive images. A region of interest (ROI) within the wing of the first image was then selected for each animal, and the average intensity within the ROI was then extracted for each animal until the time of emergence. Script data have been deposited in GitHub (https://github.com/johnewer/DMM_Analisis).

### Image Analyses: Head Roughening.

In order to quantify “head roughening,” we developed custom analysis software that analyzed the sequence of images for each animal, for all the animals within an experiment. The programs executed the following operations: 1) Allow the user to define the regions of analysis. This was done for the first image of each animal; the program then stabilized all subsequent images by using the previous image as reference. 2) Define “patches” of intensity differences within each image, and 3) calculate a “Roughness index” based either on the sum of the SD between each pixel and those immediately surrounding it (“Standard deviation” algorithm) or the sum of the perimeter of each patch (“Borders” algorithm). While these analyses were capable of generating approximate timecourses of roughness progression, we found that human inspection could determine the exact time of onset of roughening with greater accuracy. Thus, the data summarized in Fig. 2 *B* and *C* were obtained by manual detection of the start of head roughening. Scoring was blind to genotype and time of collection. Analysis software was written in Python and Java. Quantifications were done in Prism. Script data have been deposited in GitHub (https://github.com/johnewer/DMM_Analisis).

### 20E Injections.

Female white prepupae were collected at different times (ZT0 to ZT6), maintained in Petri dishes as described above, and entrained for 4 d under 12:12LD and a ca. 22 °C(L):20 °C(D) temperature cycle. On day 3, pupae were transferred from the Petri dishes to the filming disk as described above; groups were separated with small strips of red tape and humidity was maintained as mentioned above. On day 4, the operculum of all the animals was removed close to the time of lights-off. On day 5, ∼16 (collected at ZT0), 18 (ZT2), 20 (ZT4), and 22 h (ZT6) before the expected emergence, animals were injected using pulled borosilicate glass microelectrode and a PV800 pneumatic picospritzer (World Precision Instruments). Each fly was injected with ∼3.5 nl of a solution consisting of either 20E (Sigma-Aldrich) or solvent, both of which included a small volume of blue food dye, which was used to monitor the success of the injection. For 20E injections, the mixture was prepared using 2.5 µl a 10.4-mM stock solution (in ethanol) and diluted in 97.5 µL of ultrapure water (for 0.44 ng of 20 HE per injection) or 10 µl of the 20E stock solution diluted in 90 µL of ultrapure water (for 1.75 ng of 20 HE per injection); for controls, the equivalent volume of ethanol was diluted in either 97.5 or 90 µL of ultrapure water, respectively. Based on Handler (21) and Lavrynenko et al. (22), we estimate that 20E concentration was around 10 pg/animal at the time of injections. After lights-off, the temperature was maintained at the night temperature and white lights were kept off for the duration of the experiment. Time of emergence was determined using the filming setup described above.

### ETH Injections.

Female white prepupae were collected at ZT0, ZT3, and ZT6 and processed as described above for 20E injections. On day 5 and ∼9 (ZT6), 6 (ZT3), and 3 h (ZT0) before the expected time of emergence, animals were scored for their developmental proximity to emergence using the developmental markers defined by Kimura and Truman (13) and injected as described above with ∼3.5 nl of a solution consisting of either 0.5 mM of synthetic ETH (Genscript) dissolved in water (containing a small amount of blue dye) or water (with dye) alone (control). After injection, the animals were returned to the filming setup. Time of emergence was then determined using the filming setup described above. Although animals were in the dark phase when they were injected, exposure to light during the scoring and injection process did not cause premature emergence (*cf* Fig. 4*B*, controls).

### GFP Reporter Expression

Digestive systems and ovaries from pharate female flies expressing GFP under the control of *phm-GAL4* driver, and control flies were dissected on ice-cold phosphate-buffered saline and fixed in 4% paraformaldehyde for 2 h. Tissues were then washed and carefully mounted under Fluoromount-G mounting medium (Thermo Fisher Scientific) and stored in the dark until viewing. Images were taken on a Nikon C1Plus confocal microscope. Z-scans were obtained scanning preparations using a 20X objective and a z-step between 0.5 and 1.0 µm. Image projections were made using FIJI (58). Brightness and contrast adjustment were performed equally to all images using Photoshop CS5 (Adobe Systems).

### Computational Methods.

The scripts developed here, called DMM_Analisis, are available for download on GitHub.

### Statistical Analyses

Statistical analyses were carried out using Prism 6.0 (GraphPad Software Inc.).

## Supplementary Material

Supplementary File

Supplementary File

Supplementary File

## Data Availability

Script data have been deposited in GitHub (https://github.com/johnewer/DMM_Analisis).
