# Supplementary material for: The circadian clock gates Drosophila adult emergence by controlling the timecourse of metamorphosis
Source: Proc Natl Acad Sci U S A. 2021 Jun 28;118(27):e2023249118. doi: 10.1073/pnas.2023249118 (PMC8271606; doi:10.1073/pnas.2023249118)
Supplement: Supplementary File [file pnas.2023249118.sapp.pdf]

PNAS
www.pnas.org

Supplementary Information for

The circadian clock gates *Drosophila* adult emergence by controlling the timecourse of metamorphosis.

Brandon Mark*, Liliana Bustos-González*, Guadalupe Cascallares, Felipe Conejera, and John Ewer

* Co-first authors

Corresponding author: John Ewer
Email: john.ewer@uv.cl

This PDF file includes:

Supplementary Figures S1 to S6
Legends for Movies: Movie 1.m4v and Movie 2.m4v
SI References

Other supplementary materials for this manuscript include the following:

Movies: Movie 1.avi and Movie 2.avi

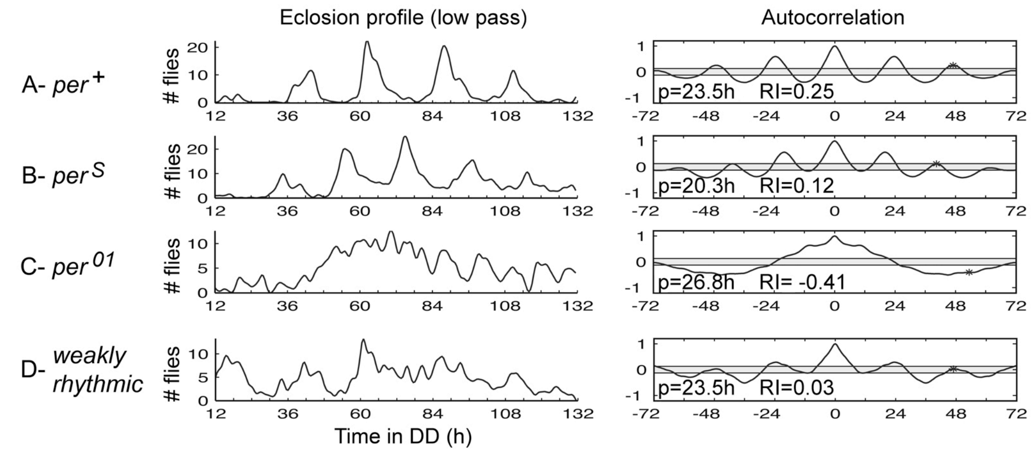

Supplementary Figure S1. **Timecourse of emergence using population assay.** The left panel shows the filtered record of the number of flies that eclose over time in constant darkness (DD) for different genotypes (genotype "weakly rhythmic" is a generic name); the right panel shows the corresponding autocorrelogram; periodicity (p, in hours) and Rhythmicity Index (RI) are indicated within the frame.

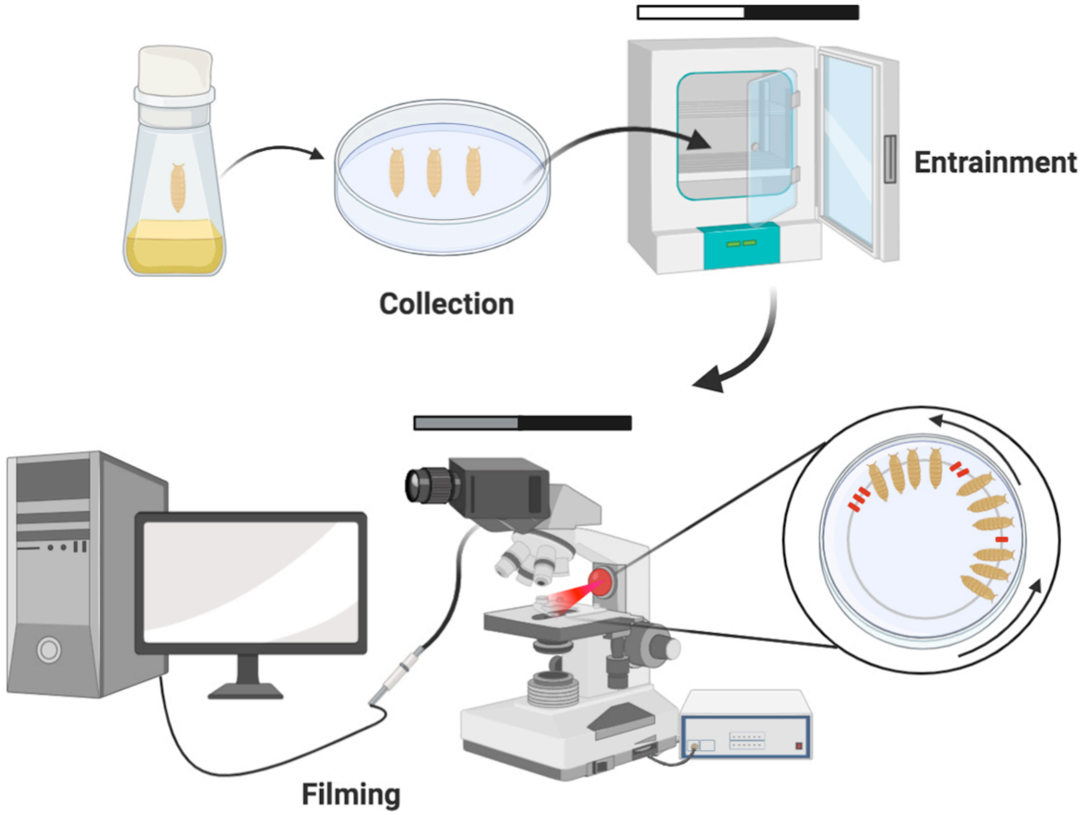

Supplementary Figure S2. Schematic of the workflow and the custom-built setup used to record the progression through metamorphosis with single animal resolution. White pupae were collected and placed on Petri dishes, and entrained with a 12h:12h L:D and temperature cycle. They were then transferred to a disc mounted on a step motor and photographed using an infrared camera as they passed under the field of view of the dissecting microscope. See the Material and Methods section for further details.

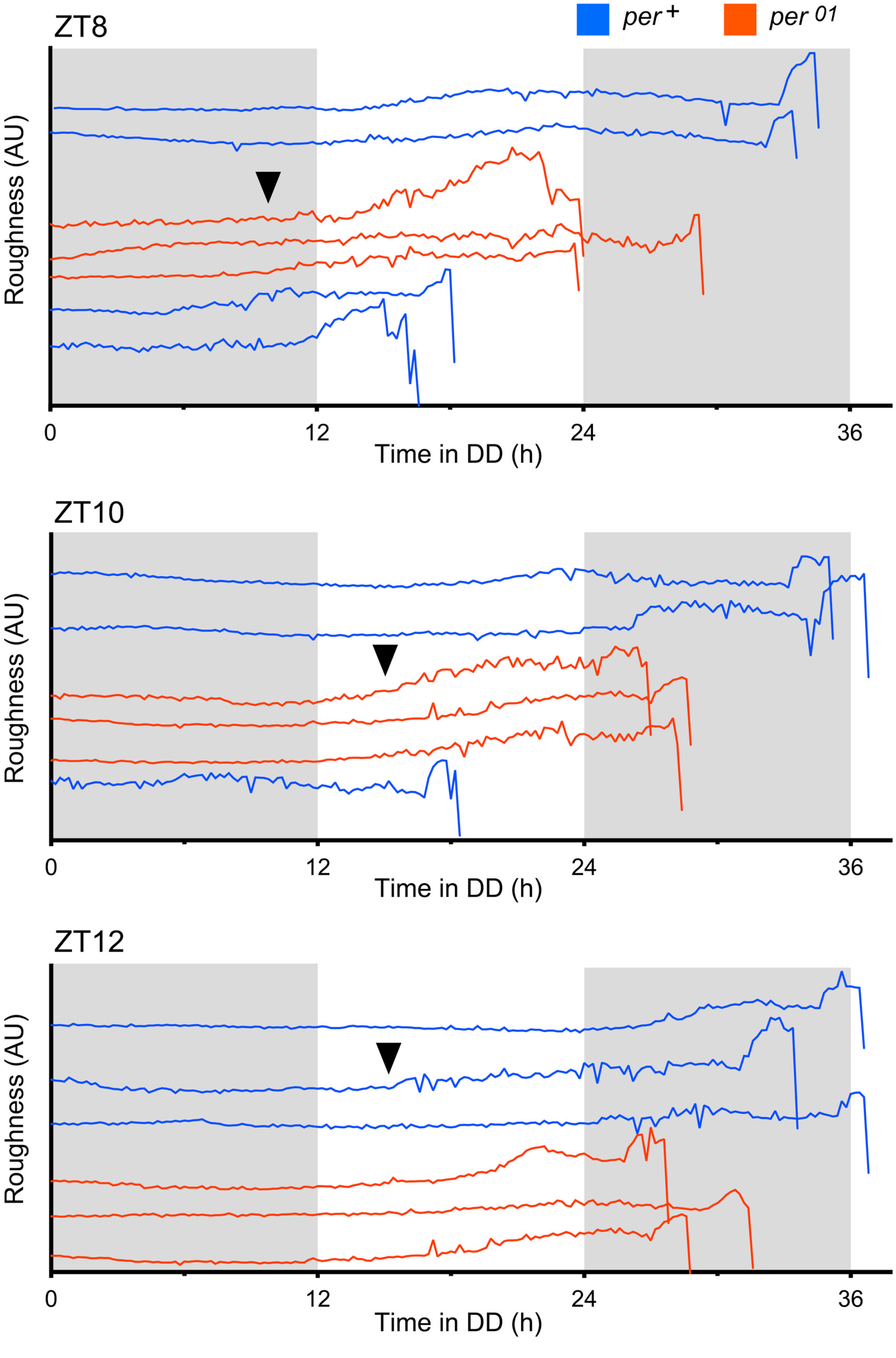

Supplementary Figure S3. Examples of the time course of head roughening for individual *per*$^{+}$ (blue lines) and *per*^{01} (red lines) flies of different ages. Each line represents a different individual fly. Sudden drop at the end of each line corresponds to the moment of emergence. Grey and white background shading represents subjective night and day, respectively. Arrowheads have been added to a few of the records to mark the approximate time at which the animal started the head roughening process (indicated by the onset of slightly higher amplitude fluctuations).

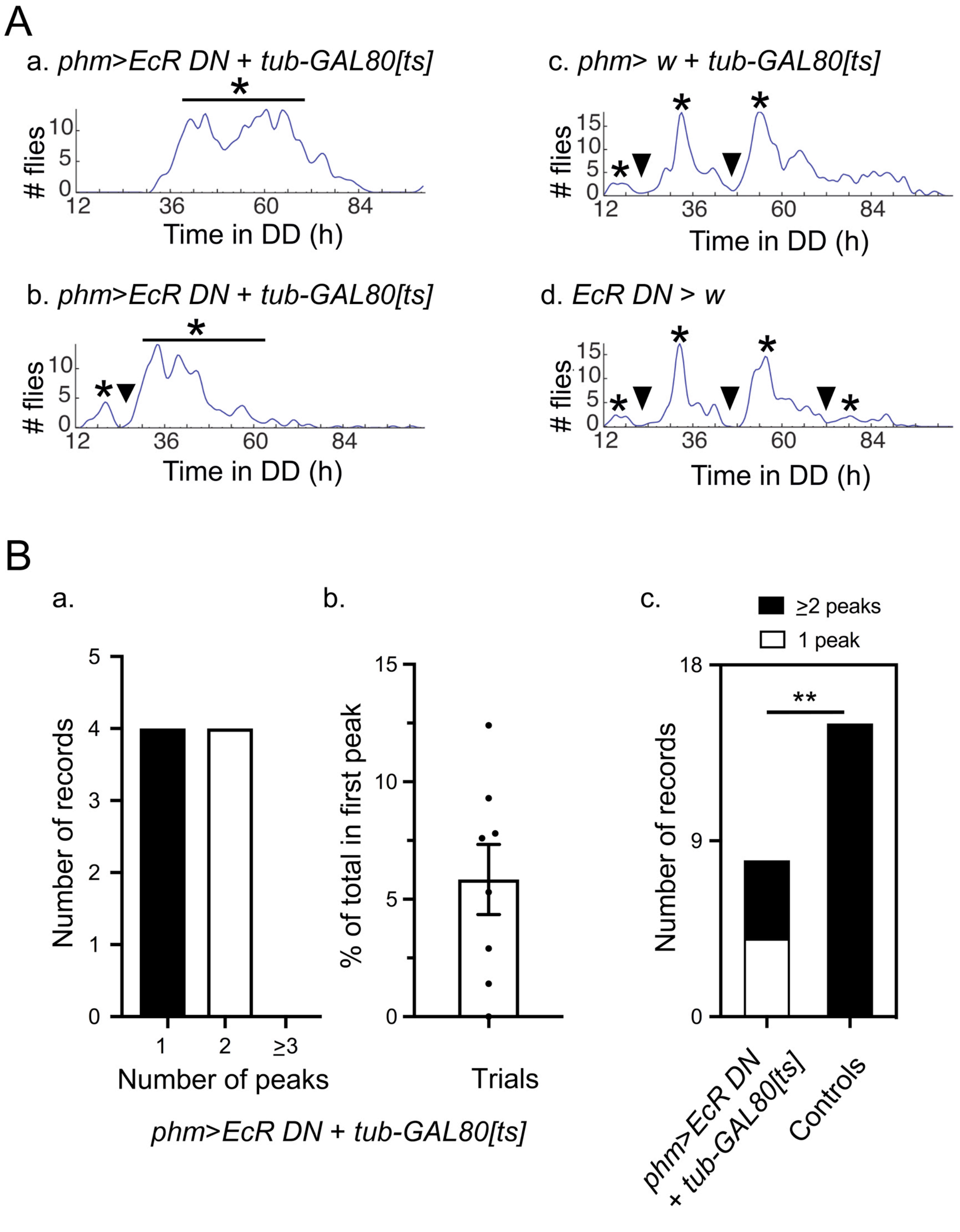

Supplementary Figure S4. Interfering with EcR signaling starting during the second half of metamorphosis disrupts the circadian rhythm of eclosion. (A) Examples of the eclosion profiles obtained when EcR DN expression in the PG was restricted to the second half of metamorphosis (a,b), and in controls (c, d). Use of the TARGET system requires these experiments to be conducted at 30°C. This causes most animals to emerge over 2-3 days, which

precludes a quantitative analysis of rhythmicity. Nevertheless, rhythmic genotypes always produce records that include peaks followed by valleys during which <5% of animals emerge, and which are separated by approximately 24h (cf., controls, c, d; peaks: asterisks; valleys: arrowheads). (B) Analysis of the number of peaks observed in the records of populations in which EcR signaling was disrupted in the PG starting during the second half of metamorphosis and in controls. (a) In 4/8 populations of *phm*>EcR DN + *tub*-GAL80[ts] all flies emerged in a single large peak, whereas in the remaining 4/8 cases emergence occurred over 2 peaks. Yet, in the latter cases the first peak represented only around 5% (5.8+1,5; average + SEM) of the total number of flies that emerged (b). The fact that this small peak always occurred first suggests that it included animals for which expression of EcR DN was not yet sufficient to interfere with EcR function, as it was always observed within the first 12h of the increase in temperature and would thus include animals that were close to emergence. For this genotype, none of the records showed more than 2 peaks. (c) Comparison of the frequency of occurrence of one and two or more peaks in the records of *phm*>EcR DN + *tub*-GAL80[ts] populations vs. controls. ** p<0,01 (Fisher's exact test analysis). N= 8 for *phm*>EcR DN+*tub*-GAL80[ts] and 15 for controls ([8] for *phm*>*w*+*tub*-GAL80[ts] and [7] for *w*>EcR DN).

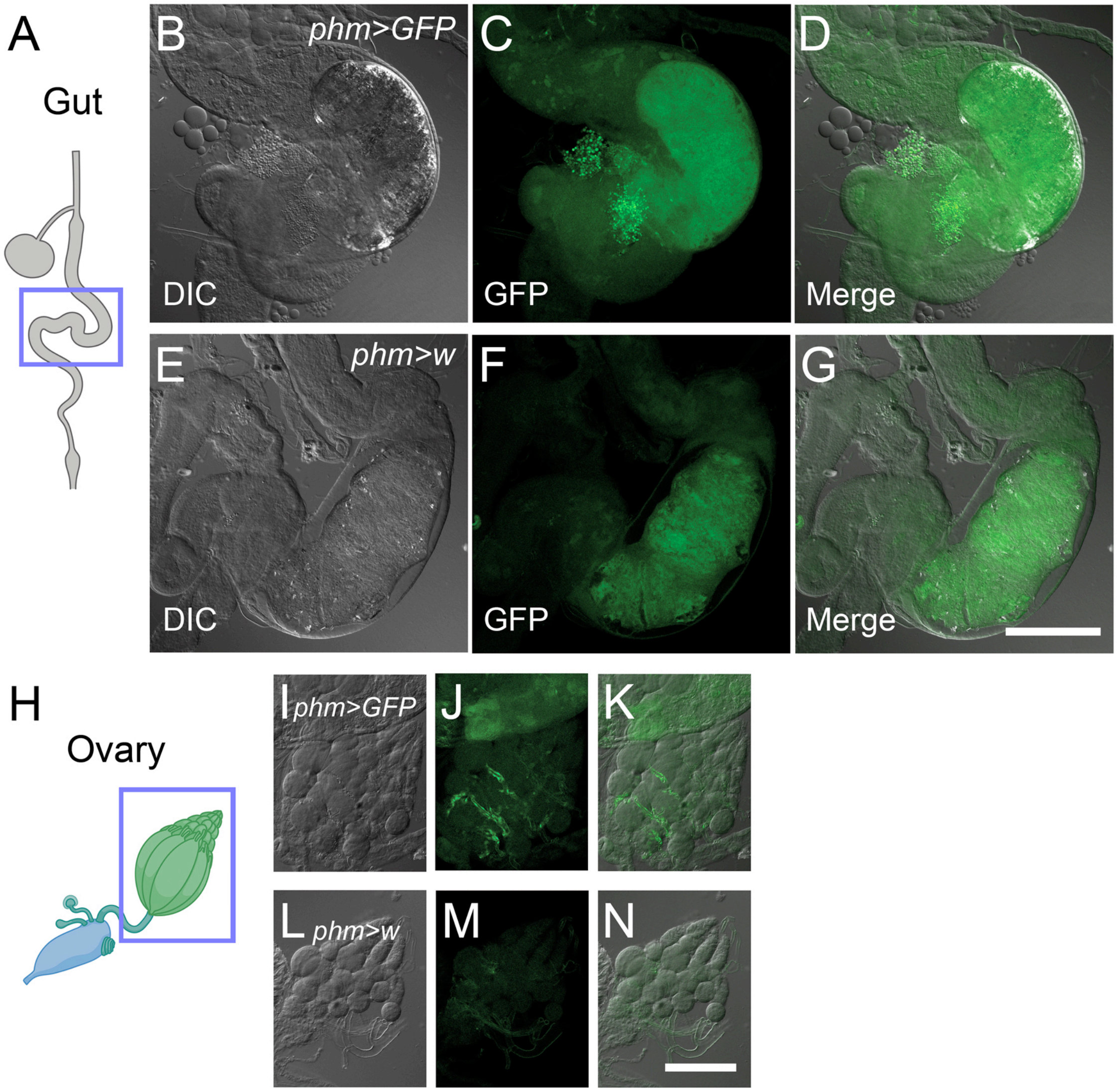

Supplementary Figure S5. Reporter expression outside of the PG driven by *phm*-GAL4 driver. (A) Schematic of the digestive system; expression in boxed area is shown in (B-G). (B-G) Representative confocal sections showing, DIC (B, E), GFP (C,F) and merged (D,G), images of the midgut of pharate female flies expressing GFP under the control of *phm*-GAL4 driver (B-D) and in controls (E-G). Mostly an autofluorescent signal was observed in the midgut region of both genotypes. (H) Schematic of the ovary; expression in boxed area is shown in (I-N). (I-N)Representative confocal sections showing, DIC (I, L), GFP (J,M), and merged (K,N), images of ovaries of pharate female flies expressing GFP under the control of *phm*-GAL4 driver (I-K) and in controls (L-N). Whether the expression detected in these tissues represents genuine sites of 20E expression is currently unknown (1). Regardless of this, tissues outside of the PG that may express the *phm*-GAL4 driver are not relevant for circadian rhythmicity since expression of EcR DN under the control of this driver did not eliminates the rhythm of adult locomotor activity (Supplementary Fig. S6)(a time by which the PG has completely degenerated (2). Scale Bar (for A-G) 40 μm; (for I-N): 30 μm.

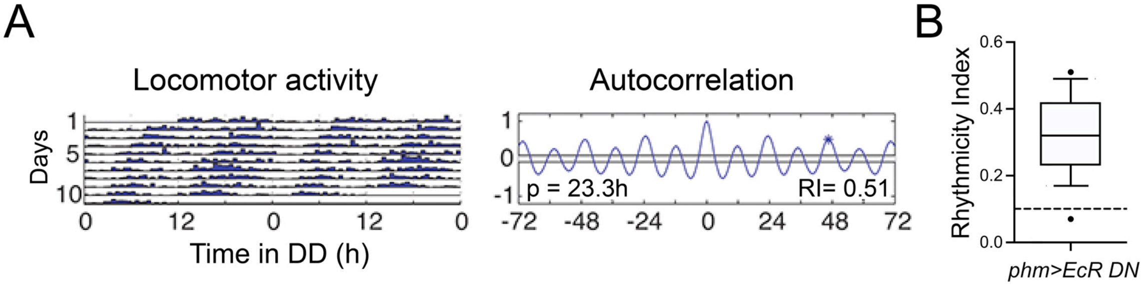

Supplementary Figure S6. Locomotor activity pattern of adult flies expressing EcR DN under the control of the *phm*-GAL4 driver. (A) Left: Representative record of adult locomotor activity pattern under DD. Right: Autocorrelation analysis with value of free-running period (p, in hours) and rhythmicity index (RI) indicated. (B) Rhythmicity Index distribution for *phm*>EcR DN flies. "Whiskers" represent 10th and 90th percentiles. Horizontal dashed line indicates RI value of 0.1, below which animals are classified as arrhythmic (3). Average RI + SEM: 0.31+ 0.028 (N=19); p<0.0001 vs. 0.10, one sample *t*-test.

Movie 1 and Movie 2.

Time course of “head roughening” for wildtype animals of the same chronological age that chose to emerge in gates separated by ca. 7h. Each movie shows the sequence of photos taken of the head of the fly during the final 1-2 days of metamorphosis. Arrowheads indicate regions of the head that first start to roughen. This occurs around 6 and 14 hours for the animals in Movie 1 and Movie 2, respectively. Note that each animal then emerged ca. 16h after the start of roughening (at around 23 and 30 hours, respectively).

SI References

1. C. C. Schwedes, G. E. Carney, Ecdysone signaling in adult *Drosophila melanogaster*. *J Insect Physiol* **58**, 293-302 (2012).
2. J.-D. Dai, L. I. Gilbert, Metamorphosis of the corpus allatum and degeneration of the prothoracic glands during the larval-pupal-adult transformation of *Drosophila melanogaster*: A cytophysiological analysis of the ring gland. *Dev. Biol.* **144**, 309-326 (1991).
3. V. Sundram *et al.*, Cellular requirements for LARK in the *Drosophila* circadian system. *J Biol Rhythms.* **27**, 183-195. doi: 110.1177/0748730412440667. (2012).
